# Lymphatic vessels are present in human saccular intracranial aneurysms

**DOI:** 10.1186/s40478-022-01430-8

**Published:** 2022-09-05

**Authors:** Nora Huuska, Eliisa Netti, Satu Lehti, Petri T. Kovanen, Mika Niemelä, Riikka Tulamo

**Affiliations:** 1 Neurosurgery Research Group, Room B410b, Biomedicum 1, Haartmaninkatu 8, 00290 Helsinki, Finland; 2 Department of Neurosurgery, Helsinki University Hospital and University of Helsinki, Topeliuksenkatu 5, 00260 Helsinki, Finland; 3 Gerontology Research Center, Faculty of Sport and Health Sciences, University of Jyväskylä, Rautpohjankatu 8, 40700 Jyväskylä, Finland; 4 Atherosclerosis Research Laboratory, Wihuri Research Institute, Haartmaninkatu 8, Biomedicum 1, Helsinki, Finland; 5 Department of Vascular Surgery, Helsinki University Hospital and University of Helsinki, Haartmaninkatu 4, 00290 Helsinki, Finland

**Keywords:** Lymphangiogenesis, Lymphatic vessels, Saccular intracranial aneurysm, Inflammation, Cerebral aneurysm

## Abstract

Saccular intracranial aneurysm (sIA) rupture leads to subarachnoid haemorrhage and is preceded by chronic inflammation and atherosclerotic changes of the sIA wall. Increased lymphangiogenesis has been detected in atherosclerotic extracranial arteries and in abdominal aortic aneurysms, but the presence of lymphatic vessels in sIAs has remained unexplored. Here we studied the presence of lymphatic vessels in 36 intraoperatively resected sIAs (16 unruptured and 20 ruptured), using immunohistochemical and immunofluorescence stainings for lymphatic endothelial cell (LEC) markers. Of these LEC-markers, both extracellular and intracellular LYVE-1-, podoplanin-, VEGFR-3-, and Prox1-positive stainings were detected in 83%, 94%, 100%, and 72% of the 36 sIA walls, respectively. Lymphatic vessels were identified as ring-shaped structures positive for one or more of the LEC markers. Of the sIAs, 78% contained lymphatic vessels positive for at least one LEC marker. The presence of LECs and lymphatic vessels were associated with the number of CD68+ and CD163+  cells in the sIA walls, and with the expression of inflammation indicators such as serum amyloid A, myeloperoxidase, and cyclo-oxygenase 2, with the presence of a thrombus, and with the sIA wall rupture. Large areas of VEGFR-3 and α-smooth muscle actin (αSMA) double-positive cells were detected in medial parts of the sIA walls. Also, a few podoplanin and αSMA double-positive cells were discovered. In addition, LYVE-1 and CD68 double-positive cells were detected in the sIA walls and in the thrombus revealing that certain CD68+ macrophages are capable of expressing LEC markers. This study demonstrates for the first time the presence of lymphatic vessels in human sIA walls. Further studies are needed to understand the role of lymphatic vessels in the pathogenesis of sIA.

**Supplementary Information:**

The online version contains supplementary material available at 10.1186/s40478-022-01430-8.

## Introduction

Lymphangiogenesis, the formation of small lymphatic vessels from pre-existing ones, plays a critical role in the regulation of immune functions and tissue fluid removal, and thus lymphatic vessels are present in almost all organ systems [1]. Impaired or excessive lymphangiogenesis has been implicated in several pathological conditions involving tissue inflammation and hypoxia [1, 2]. Large extracranial arteries such as internal carotid artery (ICA), common iliac artery, and abdominal aorta contain an adventitial network of small lymphatic vessels, which increase vascular pathologies such as atherosclerosis [3, 4]. Abdominal aortic aneurysms are also known to feature lymphangiogenesis [5]. Moreover, advanced atherosclerotic lesions in human coronary arteries and in stenotic aortic valves develop a network of lymphatic vessels [6, 7]. The current understanding is that lymphatic vessels are absent in the central nervous system, except for in the meninges, that harbour a lymphatic network [8].

Saccular intracranial aneurysm (sIA) is a saccular protrusion of a cerebral artery of an unknown origin. A sIA may rupture and lead to a subarachnoid haemorrhage with ensuing disability or death [9]. The sIAs are characterized by chronic inflammation, degenerative wall remodelling with atherosclerotic features, and thrombus formation [10–14]. Some sIA wall contain intramural neovessels, likely originating from the adventitial network of vasa vasorum [11]. Because both degenerative remodelling and chronic inflammation associate with the formation of adventitial immature neovessels in the sIA wall [11], they may also contribute to the lymphangiogenesis of the sIA wall. The cellular events of angiogenesis and lymphangiogenesis resemble each other, and they share several common growth factors [15]. Whether lymphatic vessels are present in cerebral arteries or sIAs is not known.

This study examines the presence of lymphatic endothelial cells (LECs) in human sIA walls representing various degrees of the disease development to delineate the potential role of lymphangiogenesis in the pathobiology of this disease. LECs express several specific markers, namely endothelial hyaluronan receptor 1 (LYVE-1), podoplanin, vascular endothelial growth-factor receptor 3 (VEGFR-3), and prospero homeobox protein 1 (Prox1) [16], and they were studied by immunohistochemical staining methods in this series of samples of human sIA walls. Importantly, as lymphangiogenesis already provides a therapeutic target in various human pathologies [16], it would serve as a relevant area of interest in the research of potential treatments for sIAs.

## Materials and methods

### Samples of saccular intracranial aneurysms

A previously published sIA series [11–13, 17–19] of 36 sIA samples (16 unruptured and 20 ruptured) were studied. The sIA samples were resected after surgical clipping at the Department of Neurosurgery, Helsinki University Hospital (HUH), Helsinki, Finland. The samples were immediately snap-frozen in liquid nitrogen after harvesting and stored at  − 80 °C. For immunohistochemical and immunofluorescence stainings, the frozen samples were embedded in Tissue-Tek (Sakura, Alphen aan den Rijn, the Netherlands) and cryosectioned at 4 µm. Clinical data were collected from the patients’ medical records and sIA dimensions were obtained from preoperative computed tomography angiography images. The HUH Ethics Committee approved this study.

### Basic characteristics of the aneurysm walls

The sIAs were classified into categories A–D according to the characteristics of their walls: type A (9/36; 25%), type B (12/36; 33%), and type C (11/36; 31%) [11]. Only two samples representative of wall type D (2/36; 6%) were present and therefore they were excluded from further analyses. The following wall classification criteria were originally published by Frösen et al., 2004 [20]: wall type A displays a wall with intact endothelium and an organized layer of smooth muscle cells (SMCs), type B displays a thickened wall and a disorganized layer of SMCs, type C displays a hypocellular wall with either myointimal hyperplasia or organized thrombus, and type D displays a very thin hypocellular wall with organized thrombus. This sIA series has also been analysed earlier for inflammatory and lipid characteristics [11–13, 18, 19].

### Immunohistochemistry and immunofluorescence stainings

For immunohistochemical stainings, the histological sections were fixed with ice-cold acetone for 3 min, and sequentially incubated with the EnVision Kit’s blocking reagent (Dako, Santa Clara, CA, USA) and 3% normal horse serum (NHS; Vector, Burlingame, CA, USA) at room temperature (RT) for 30 min. The primary antibodies against lymphatic vessels i.e. against the studied LEC-markers: podoplanin, Prox1, LYVE-1, and VEGFR-3 (Additional file 1: Table S1) were diluted to the buffer solution from the kit and incubated on the sections for 60 min at RT. The secondary detection was performed with the EnVision Kit’s horseradish peroxidase reagent (Dako) for the mouse primary antibodies and with the anti-goat secondary antibody diluted 1:200 for the goat primary antibodies according to the manufacturer’s protocol. For the detection of the positive signal, the sections were incubated in diaminobenzidine for 4 min at RT. Finally, the sections were background stained with Mayer’s haematoxylin (Sigma-Aldrich, St. Louis, MO, USA) or with Lillie’s Modification (Dako) and embedded in a mounting aqueous medium (Faramount, Dako). An irrelevant mouse monoclonal antibody (IgG1 or IgG2a, depending on the subclass of primary antibody; Serotec, Oxford, UK) served as a substitute for the primary antibody in negative controls in the podoplanin, LYVE-1, and VEGFR-3 stainings. In the Prox1 stainings, the primary antibody was omitted in the negative controls. Freshly frozen human tonsil tissue served as a positive control in both immunohistochemical and immunofluorescence stainings.

For immunofluorescence double stainings the frozen sIA sections were fixed and blocked as described above. The primary antibodies against podoplanin or LYVE-1 (Additional file 1: Table S1) were diluted to the buffer solution and incubated on sections for 60 min at RT. The secondary detection was performed by incubating the sections with Alexa fluor 488 (green) F(ab’)2 fragments of goat anti-mouse or rabbit anti-goat IgG antibodies (Thermo Fisher Scientific, Eugene, OR, USA), respectively, at RT for 20 min. Thereafter, the sections were re-blocked and incubated in primary antibodies against α-smooth muscle actin (αSMA) or CD68 (Additional file 1: Table S1). The secondary detection of CD68 was performed by incubating the sections with Alexa fluor 594 (red) F(ab’)2 fragments of rabbit anti-goat IgG antibody (Thermo Fischer Scientific). Finally, the sections were stained for nuclei with DAPI (Sigma-Aldrich) and embedded in fluorescence mounting medium (Faramount, Dako). The primary antibody was omitted in the negative controls.

### Analysis of immunohistochemical and immunofluorescence stainings

The immunohistochemical and immunofluorescence stainings were scanned using 3DHISTECH Pannoramic 250 FLASH II digital slide scanner (Budapest, Hungary). Positive stainings for LYVE-1, podoplanin, VEGFR-3, and Prox1 and their histological locations were analysed semiquantitatively from all 36 stained sIAs.

**Fig. 1:**
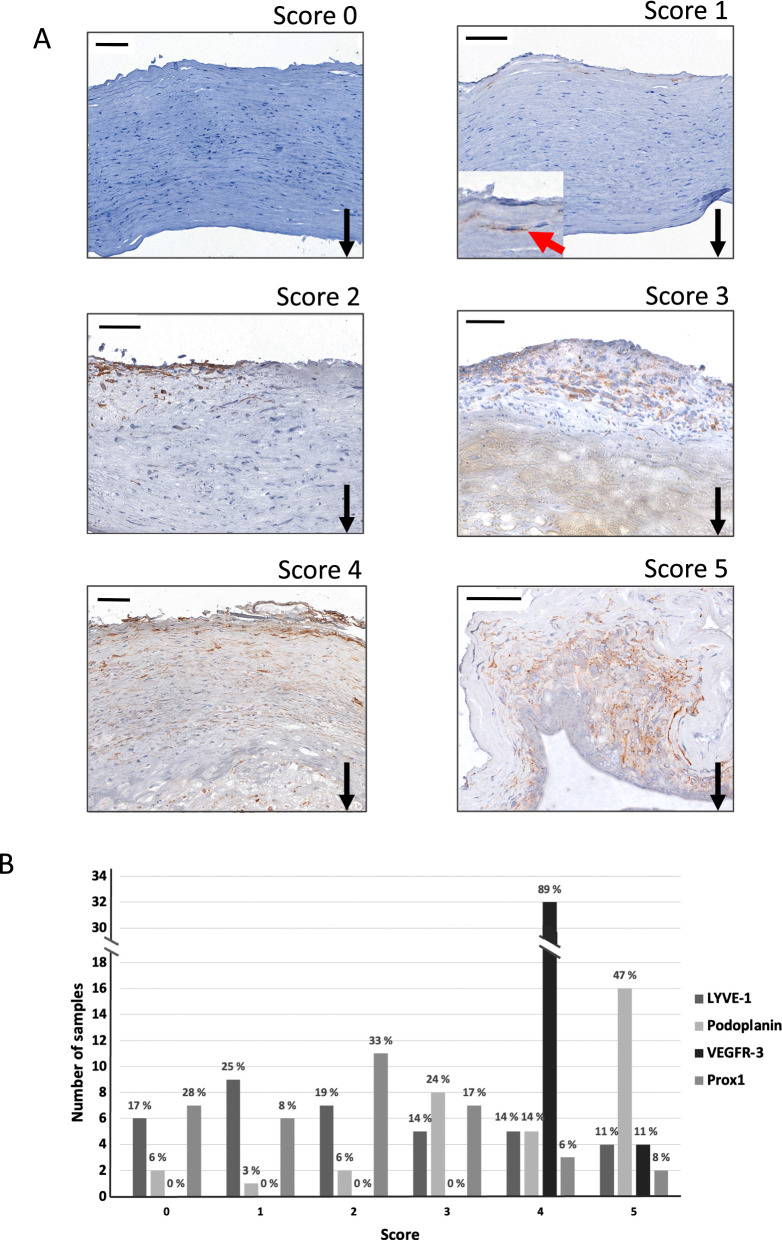
Representative images of saccular intracranial aneurysm (sIA) walls presenting immunohistochemical staining for LYVE-1 scores 0, 1, 2, 3, 4, and 5 (**A**). Score 0 represents a wall with no positive staining, score 1 a wall with a few positive cells, score 2 a wall with 1–2 clusters of positive cells, score 3 a wall with several clusters of positive cells, score 4 a wall with scattered positive cells throughout the entire wall, and score 5 a wall with a widespread area of positive staining. Black arrows point down towards the lumen. Intracellular LYVE-1 is indicated with a red arrow and shown as an inset in the image for score 1. Positive staining is brown. Haematoxylin background staining. Scale bar: 100 μm. (**B**) Distribution of the 36 sIA walls into LYVE-1, podoplanin, VEGFR-3, and Prox1 scores 0–5. Percentages represent the proportion of the scores within the staining

The samples were scored semiquantitatively as 0–5 depending on the extent and location of the positively stained area (Fig. 1A) using the highest score for each sample. The presence and the number of positively stained ring-shaped structures were considered as cross-sections of lymphatic vessels and counted in each sample of LYVE-1, podoplanin, Prox1, and VEGFR-3 stainings.

**Fig. 2:**
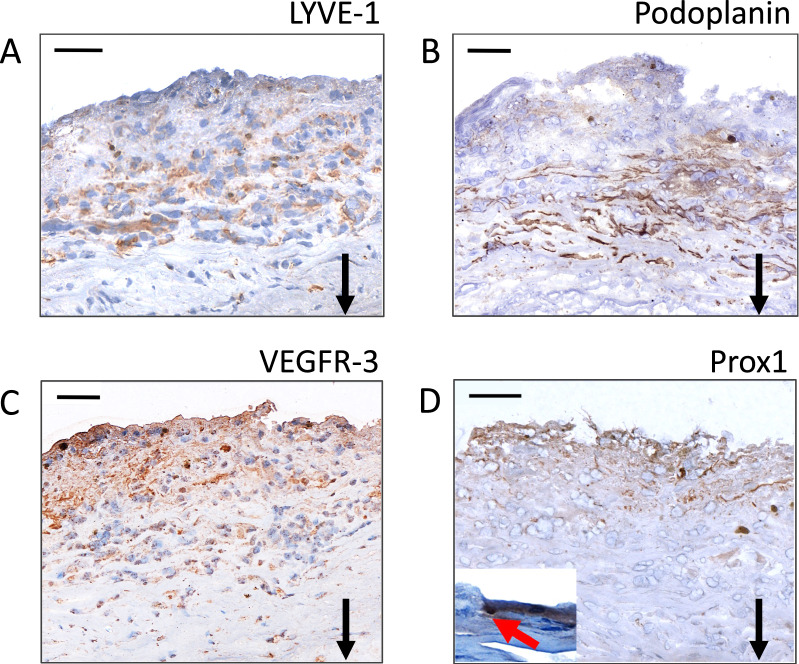
Representative images of positive immunohistochemical stainings for LYVE-1 (**A**), podoplanin (**B**), VEGFR-3 (**C**), and Prox1 (**D**) in a single unruptured saccular intracranial aneurysm. The images show the same area of the aneurysm wall in different sections. A positively stained nucleus is indicated with a red arrow and shown as an inset in **D**. Black arrows point down towards the lumen. Scale bar: 50 μm. Positive staining is brown. Haematoxylin background staining

The semiquantitative analysis of LYVE-1, podoplanin, Prox1, and VEGFR-3 and the presence and the number of positively stained ring-shaped structures, i.e. lymphatic vessels were compared to sIA rupture status, sIA wall degeneration (wall type), presence of thrombus, and markers for lipids, inflammation, and angiogenesis as defined in Additional file 1: Table S1 and in our previous studies [11–13, 18, 19]. The percentual proportion of the thrombus area in the sIA wall was defined by measuring the area of the thrombus in the sample section using the 3DHISTEC Slide viewer area tool and dividing it by the total sIA wall area (including the thrombus). The overlap of a LYVE-1-positive area with areas positive for podoplanin, Prox1, VEGFR-3, or angiogenesis (CD34 staining, [11]) on consecutive sections was defined as complete, partial, or absent, as demonstrated in Fig. 2. The presence and location of double-positive staining for podoplanin and αSMA, and for LYVE-1 and CD68 were determined in scanned immunofluorescence double stainings.

### Statistics

Data analysis was performed using the IBM SPSS Statistics Software, version 27. For categorical variables, proportions were calculated, and Fisher’s exact test (F) was used. For continuous variables, Kruskal–Wallis (KW) multiple comparison test, Mann–Whitney-U (MWU) test, and Spearman (S) correlation test were used. P-values < 0.05 were considered statistically significant.

## Results

### Lymphatic endothelial cell (LEC) markers and their staining patterns in the sIA walls

LYVE-1-, podoplanin-, VEGFR-3-, and Prox1-positive staining were detected in the majority (83%, 94%, 100%, and 72%, respectively) of the 36 sIA walls both extracellularly and intracellularly (Fig. 1B). Distributions of the LYVE-1, podoplanin, VEGFR-3, and Prox1 stainings in the sIA walls are described in Table 1. A few Prox1- stained nuclei were detected in 12/36 samples, where their number varied from 1 to 5 per sample. The stainings for LYVE-1, podoplanin, and Prox1 localized primarily in the adventitial side of the sIA walls, whereas VEGFR-3-positive cells were detected throughout the walls (Fig. 2). The intensity and extent of these stainings were analysed semiquantitatively with scores of 0–5, as defined in Fig. 1B. Within the thrombi, positive stainings for all four LEC markers were observed, and their extent ranged from a few positive cells to large diffuse areas (Additional file 2: Figure S1).

**Table 1:** Positive staining patterns of lymphatic endothelial cell (LEC) markers in the saccular intracranial aneurysm (sIA) walls

LEC marker*	sIAs positive for LEC marker	Location in the sIA wall	Cellular /extracellular location	sIAs with lymphatic vessels	Staining pattern within thrombus	Colocalization with LYVE-1 staining in consecutive sections
LYVE-1	30/36 (83%)	Adventitial; variation in the extent of staining	Cellular and extracellular	19/36 (52%)	13/17 (76%) single positive cells; lymphatic vessels in2/17 (12%)	–
Podoplanin	32/34 (94%)	Adventitial; large positively stained areas	Cellular and extracellular	23/34** (68%)	14/17 (82%) single positive cells and large, diffuse positively stained areas; lymphatic vessels in 3/17 (18%)	Complete in 8/29 (28%) and partial in 18/29 (62%) samples positive for both stainings
VEGFR-3	36/36 (100%)	Scattered positive cells in all samples, wide positively stained adventitial areas in 4/36 (11%) samples	Cellular and extracellular	11/36 (30%)	17/17 (100%) large positively stained areas; lymphatic vessels in 0/17 (0%)	Wide positively stained areas in the adventitial side of the wall in 4/36 (11%) samples colocalized completely with the LYVE-1-positive staining
Prox1	26/36 (72%)	Adventitial; small positively stained areas	Cellular and extracellular;positive nuclei in 12/36 samples	8/36 (22%)	12/17 (71%) single positive cells and small, diffuse positively stained areas; lymphatic vessels in 0/17 (0%)	Complete in 2/23 (9%) and partial in 13/23 (56%) samples positive for both stainings

*LYVE-1 (lymphatic vessel endothelial hyaluronic acid receptor-1), VEGFR-3 (vascular endothelial growth factor receptor 3), Prox1 (prospero-related homeobox 1)

**In the podoplanin staining, the hematoxylin background staining was faded in two samples by the time of analysis

Therefore, the localization of the positive podoplanin staining could not be analyzed and the samples were discarded from the analysis

**Fig. 3:**
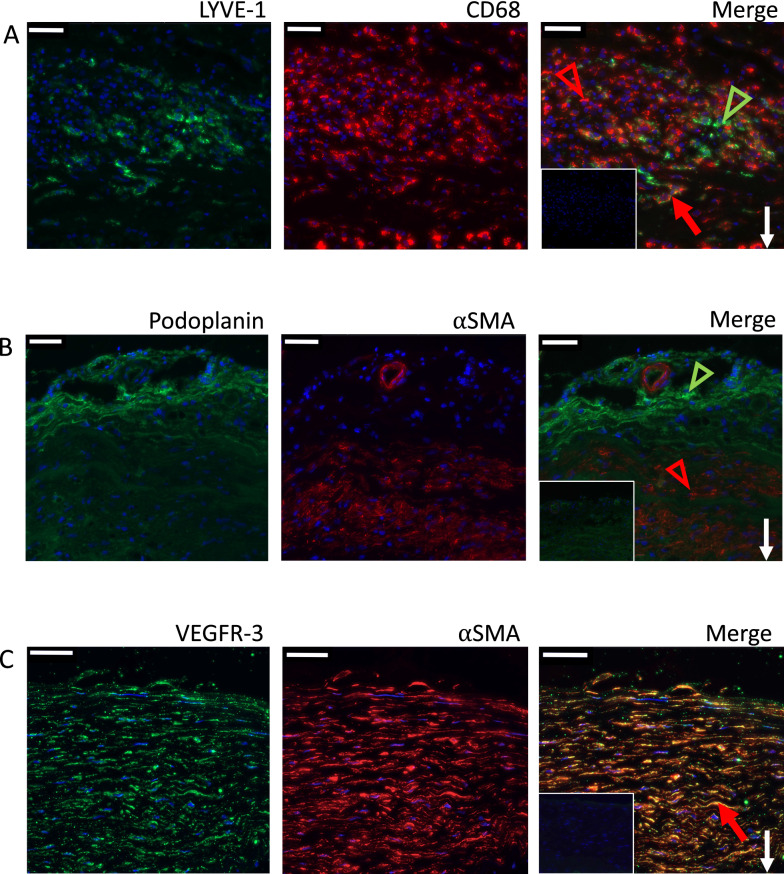
Representative images of immunofluorescence double stainings for **A** LYVE-1 (green) and CD68 (red), **B** podoplanin (green) and αSMA (red), and **C** VEGFR-3 (green) and αSMA (red) in three saccular intracranial aneurysm walls. Double-positive staining (yellow) is indicated with a red arrow in panels **A** and **C**. LYVE-1- and podoplanin-positive stainings (green) are indicated with green arrowheads in panels **A** and **B**, respectively. Positive staining for αSMA (red) is indicated with red arrowheads in panels **A** and **B**. Green ring-shaped structure positive for podoplanin and negative for αSMA demonstrates a lymphatic vessel and red ring-shaped structure positive for αSMA and negative for podoplanin demonstrates a vascular neovessel in panel **B**. The negative controls are shown as insets. White arrows point down towards the lumen. Scale bar: 50 μm

The sIA walls contained scattered podoplanin-positive spindle-shaped cells that were located in the medial part of the wall and resembled smooth muscle cells by shape and location. In immunofluorescence double stainings of podoplanin and α-smooth muscle actin (αSMA) in 6 selected sIA samples, however, only a few smooth muscle cells in the sIA wall colocalized with podoplanin-positive stainings (Fig. 3. The VEGFR-3 positive stainings were primarily located in single positive spindle-shaped cells throughout the wall (score 4) also resembling smooth muscle cells (Fig. 2). The immunofluorescence double staining of VEGFR-3 and αSMA in 5 samples revealed large areas of double-positively stained cells in the medial parts of the sIA walls (Fig. 3), indicating that smooth muscle cells in the sIA walls widely express VEGFR-3. In the immunofluorescence double staining of LYVE-1 and CD68, double-positive cells were also detected in the 6 studied sIA walls and in the thrombus (Fig. 3), indicating that CD68-positive cells in the sIA wall and in thrombus expressed LYVE-1.

### LEC-positive lymphatic vessels in the sIA walls

**Fig. 4:**
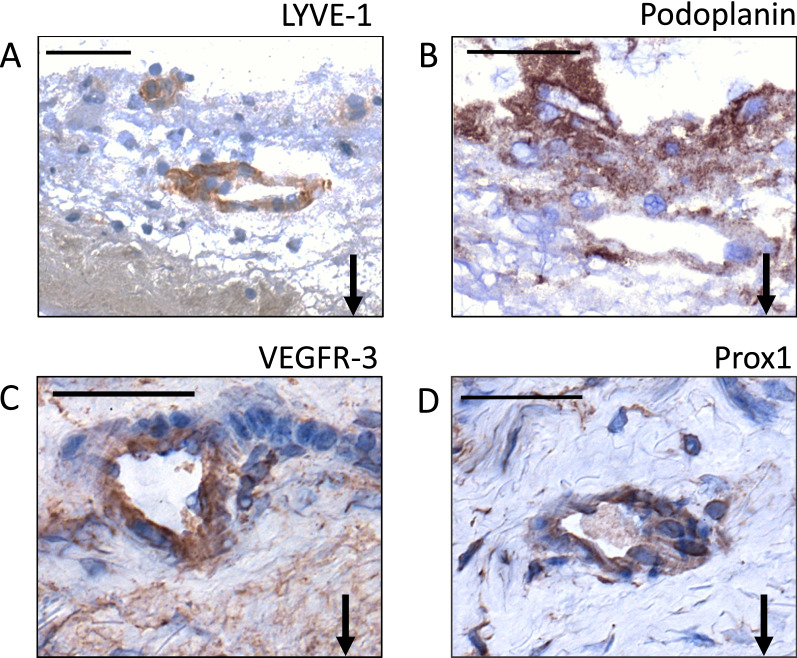
Representative images of ring-shaped structures positive for LYVE-1 (**A**), podoplanin (**B**), VEGFR-3 (**C**), and Prox1 (**D**), i.e. lymphatic vessels in four saccular intracranial aneurysm walls. Black arrows point down towards the lumen. Scale bar: 50 μm. Positive staining is brown. Haematoxylin background staining

Ring-shaped structures staining positively for one or more LEC-specific antigens enabling the identification of lymphatic vessels were detected in 22 to 68% of the samples, depending on the LEC marker (Fig. 4, Table 1). 78% of the samples contained lymphatic vessels positive for at least one LEC marker. The lymphatic vessels stained most distinctly in the LYVE-1 staining but were most frequent in number in the podoplanin stained samples. Prox1- and VEGFR-3-positive lymphatic vessels were rare and stained weakly, and therefore were challenging to detect. None of the lymphatic vessels colocalized with the CD34-positive vascular neovessels. The luminal endothelium in the sIA walls did not stain positively for any of the LEC markers. We could not identify any lymphatic vessel, which had been positive for all the studied four LEC markers. However, in consecutive sections, some lymphatic vessels positive for three of the markers used, i.e., LYVE-1, podoplanin, and VEGFR-3, were detected.

### Associations of LEC markers with other sIA-related markers

Several correlations between the expression of the LEC markers and the presence of lymphatic vessels with other sIA-related markers were detected (Table 2).

**Table 2:** Associations of lymphatic endothelial cell (LEC) marker scores and presence of lymphatic vessels in the saccular intracranial aneurysm (sIA) walls with sIA-related variables

	LEC marker scores**			Presence of lymphatic vessels				
Variable*	LYVE-1	Podoplanin	Prox1	LYVE-1	Podoplanin	Prox1	VEGFR-3	Any LEC+ vessels
*Clinical variables*								
Sex†	NS	NS	NS	NS	NS	NS	NS	NS
Smoking, current†	NS	NS	NS	NS	NS	NS	0.027	NS
Presence of multiple IAs†	0.012	0.031	NS	NS	NS	NS	0.002	NS
*Characteristics of the sIA wall*								
Rupture†	NS	NS	NS	0.042	NS	NS	NS	NS
Wall type†	NS	NS	NS	NS	NS	NS	NS	NS
Presence of thrombus†	NS	NS	NS	0.010	0.041	NS	0.010	NS
Area of thrombus‡	0.025	NS	NS	NS	NS	NS	NS	NS
Presence of CD34 + or CD31 + neovessels†	NS	NS	NS	NS	NS	NS	NS	0.011
*Presence of cells in the sIA wall §*								
Mast cells‡	NS	NS	NS	NS	NS	0.031	NS	NS
CD3 + T lymphocytes‡	NS	NS	NS	NS	NS	NS	NS	NS
CD68 + macrophages‡	NS	NS	NS	NS	NS	0.030	NS	0.030
CD163 + macrophages‡	0,046	0,045	NS	NS	NS	NS	0.033	NS
Red blood cells†	NS	NS	0.003	NS	NS	0.003	0.028	NS
*Presence of inflammatory markers in the sIA wall*								
SAA accumulation†	NS	NS	0.009	0.013	NS	0.009	0.018	0.036
MPO expression †	NS	NS	0.020	0.008	0.032	0.012	0.030	0.043
COX2 expression †	NS	NS	NS	NS	NS	NS	0.015	NS
MMP-9 expression †	NS	0.021	NS	NS	NS	NS	NS	NS
*Area (%) of lipid accumulation markers in the sIA wall*								
ORO+ ‡	NS	NS	NS	NS	NS	NS	NS	NS
ApoA-1+ ‡	NS	NS	0.002	NS	NS	NS	NS	NS
oxLDL+ ‡	NS	NS	0.018	0.049	NS	0.031	NS	NS
Adipophilin+ ‡	NS	NS	NS	NS	NS	NS	NS	NS

*P*-values < 0.05 were considered statistically significant.

*LYVE-1 (lymphatic vessel endothelial hyaluronic acid receptor-1), VEGFR-3 (vascular endothelial growth factor receptor 3), Prox1 (prospero-related homeobox 1), CD (cluster of differentiation), SAA (serum amyloid A), MPO (myeloperoxidase), COX2 (cyclo-oxygenase 2), MMP-9 (matrix metalloproteinase 9), ORO (Oil-Red O, i.e. neutral lipids), ApoA-1 (apolipoprotein A-1), LDL (low density lipoprotein).

**In the immunohistochemical staining for VEGFR-3, 32/36 sIA walls were score 4 and 4/36 sIA walls were score 5, and therefore, the VEGFR-3 score was excluded from further analysis.

†Fisher’s exact test was used.

‡Kruskal–Wallis test was used for LEC-marker scores and Mann–Whitney-U test was used for lymphatic vessels

§Presence of mast cells, number of CD3+ , CD68+ , and CD163+ cells, and glycophorin score (semiquantitative)

The LYVE-1-positive vessels were associated with the sIA wall rupture, but no association between sIA wall type and LEC-markers or lymphatic vessels was found. Patients with multiple sIAs showed more often LEC-markers and lymphatic vessels than patients with single sIAs.

**Fig. 5:**
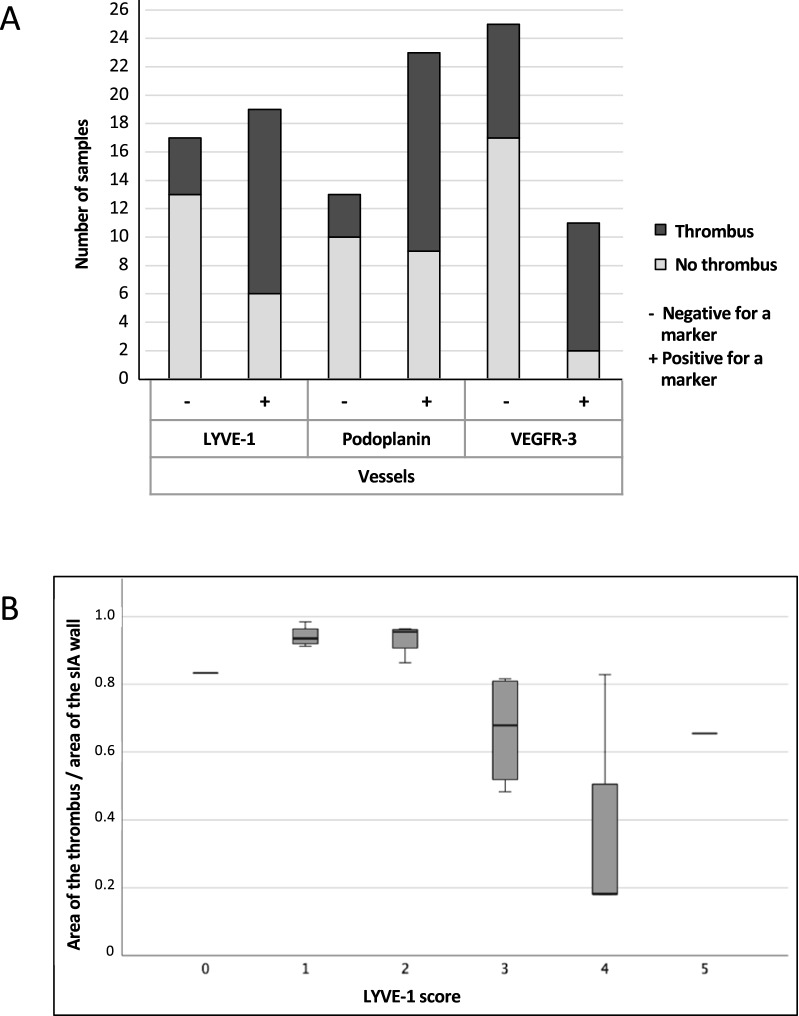
Bar grafts of the distribution of thrombus in saccular intracranial aneurysm wall samples presenting positive staining for LYVE-1, podoplanin, and Prox1 vessels vs. no staining for LYVE-1, podoplanin, and Prox1 vessels (**A**). Association of LYVE-1 score with the percentual proportion of the thrombus in the sIA wall (**B**)

The presence of lymphatic vessels was associated with the presence of a thrombus in the sIA wall (Fig. 5A). However, LYVE-1 score was associated negatively with the proportion of the thrombus area in the sIA wall; when the proportion of the thrombus area in the sIA wall increased, the extent of the LYVE-1-positive area decreased in the sIA wall (Fig. 5B).

The presence of lymphatic vessels and extent of Prox1 staining showed an association with the expression of two inflammatory markers in the sIA wall: serum amyloid A (SAA) and myeloperoxidase (MPO). Another inflammatory marker in the sIA wall that was associated positively with lymphatic vessels was cyclo-oxygenase 2 (COX2). In addition, an association between podoplanin and the presence of matrix metalloproteinase 9 (MMP-9) was detected. The sIA walls with Prox1-positive vessels showed higher numbers of CD68+ macrophages and contained mast cells more often than sIAs without Prox1-positive vessels. Moreover, the samples with VEGFR-3-positive vessels showed higher numbers of CD163+ macrophages, than samples without VEGFR-3-positive vessels. Neither did the lymphatic vessels or LEC-markers associate with the number of CD3+ lymphocytes. Furthermore, LYVE-1 and podoplanin scores were associated positively with the number of CD163+ cells.

The presence of Prox1-positive lymphatic vessels and the Prox1 score were positively associated with the extent of oxidized LDL-positive staining. In addition, the Prox1 score was positively associated with the apolipoprotein A1-positive staining. Moreover, the Prox1 score and the presence of Prox1- and VEGFR-3-positive vessels showed an association with the extent of glycophorin A-positive staining in the sIA wall, suggesting their association with the accumulation of red blood cell debris in the sIA wall.

## Discussion

Our study is the first to demonstrate the presence of lymphatic vessels in human sIA walls, pathologic outpouching of intracranial arteries. LEC markers were present in the majority of the studied 36 sIA walls and there was a great variation in the extent of their presence between the samples, suggesting that some sIA walls showed more extensive lymphangiogenesis than others. Similar to other vascular pathologies [3–5, 21, 22], the LEC-markers were present both extracellularly and intracellularly, and they were typically localized in the adventitial side of the sIA wall. The sIA wall contained also ring-shaped structures, containing an endothelium positive for the studied LEC-markers, and thus were considered to represent small lymphatic vessels.

The sIA wall is characterized by changes resembling atherosclerosis, namely the accumulation of oxidized lipids and inflammatory cells, and by degenerative wall remodelling and thrombus formation [10–14]. In this study, we found that the presence of lymphatic vessels associated with atherosclerotic changes such as the presence of thrombus and accumulation of oxidized LDL. In large atherosclerotic extracranial arteries, such as the internal carotid artery, and the abdominal and iliac segments of the aorta, the numbers of small lymphatic vessels have been found to be higher than in the corresponding non-atherosclerotic arterial segments [3, 4]. However, no association between the sIA wall type and LEC-markers or lymphatic vessels was found in this study, suggesting that lymphangiogenesis may not affect the sIA degenerative wall remodelling.

In the sIA walls, the presence of lymphatic vessels was also associated with the accumulation SAA and the expression of MPO. Such associations have not been reported in atherosclerotic arteries and require further investigation. In this study, the presence of thrombus did associate with the presence of lymphatic vessels in the adjacent sIA wall. In addition, positive staining for all studied LEC-markers and a few lymphatic vessels were also detected within the luminal thrombus suggesting the presence of lymphangiogenic factors also in the thrombus. Of note, in studies of lymphangiogenesis in other types of aneurysms, e.g. abdominal aortic aneurysms, the presence of thrombus and its relationship with the studied LEC-markers has not been described [5].

Prox1 is a transcription factor and thus is usually located in the nucleus [23], but in the sIA walls, it was also detected in the cytoplasmic compartment. Although studies of Prox1 in vascular pathobiology are scarce, cytoplasmic Prox1 has been detected earlier in human lens cells and thyroid and gastric cancers [24–26]. It remains unknown, whether and how Prox1 expression or localization within the cell changes during the LEC maturation. While Prox1 and VEGFR-3 are expressed in LECs from the beginning of the lymphangiogenesis, podoplanin is expressed at the end of the development of lymphatic vessels, and thus serves as a marker of the late stages of lymphangiogenesis [27]. LYVE-1 expression is decreased on lymphatic pre-collectors and absent from collectors, but it remains high in lymphatic capillaries [28]. In the studied sIAs, the expression of LEC markers differed between the sIAs, suggesting variability in phases of lymphangiogenesis and aneurysm progression. Further studies are required to discover, how the expression of LEC markers change during the sIA wall development and progression.

In this study, the presence of lymphatic vessels was associated with the number of CD163 + and CD68 + macrophages. Also, double-positive cells for LYVE-1 and CD68 were detected in the immunofluorescence stainings. LYVE-1, podoplanin, and VEGFR-3 are not exclusively expressed on LECs, but they are also expressed on a variety of other cell types. In fact, in pathological lymphangiogenesis, macrophages can transdifferentiate into lymphatic endothelial cell progenitors and stimulate the pre-existing local lymphatic endothelial cells to divide via the release of pro-lymphangiogenic factors [29]. Only little is known about the function of LYVE-1-positive macrophages in human artery walls; in the arterial walls of human abdominal skin and in the umbilical cord, LYVE-1-positive macrophages have been detected to maintain the arterial tone by controlling the expression of collagen in vascular smooth muscle cells [30]. The specific functions of LYVE-1-positive macrophages in the sIA wall are yet to be determined.

VEGFR-3 is reported to be expressed on smooth muscle cells in both atherosclerotic and healthy human major arteries [31]. Also in atherosclerotic lesions, podoplanin-positive macrophages and smooth muscle cells have been discovered, and their proportion increases with the progression of atherosclerotic lesions [32]. In the sIAs, the immunofluorescence double-staining of VEGFR-3 with αSMA revealed large areas of VEGFR-3-positive smooth muscle cells. However, the immunofluorescence double-stainings of podoplanin with αSMA revealed only a small number of double-positive cells in the sIA wall.

### Limitations of the study

Firstly, in the sIA surgery, since the base is covered by a clip, only a fundus of sIA can be resected. Secondly, because of the small sample size, there was no possibility to prepare several sections throughout the sample. Thus, our conclusions are restricted only to single section stainings. Thirdly, our findings in patients represent only a single point in the timeline of the pathogenesis of the sIA development: the moment of sample collection. Thus, the role of lymphangiogenesis in the sIA long continuum of the progression of the disease remains unexplored in the patients who had undergone resection surgery of the sIA.

## Conclusions

This is the first study demonstrating the presence of lymphatic vessels in the human sIA wall revealing lymphangiogenic potential of the sIAs. However, our data do not clarify the exact function of the lymphatic vessels in the sIA wall or, whether lymphangiogenesis is beneficial or harmful for the sIA. For this purpose, further experimental in vivo studies are warranted on sIA models.

## Supplementary Information

**Additional file 1: Table S1.**

**Additional file 2: Fig. S1.** Representative images of thrombus in the saccular intracranial aneurysm (sIA) presenting immunohistochemical staining for LYVE-1 (**A**), podoplanin (**B**), VEGFR-3 (**C**), and Prox1 (**D**). Ring-shaped structures of immunohistochemical staining positive for LYVE-1 (**E**) and podoplanin (**F**), i.e., lymphatic vessels, in the sIA thrombus. The negative controls are shown as insets. Black arrows point down towards the lumen. Scale bar: 50 μm. Positive staining is brown. Haematoxylin background staining.

## Data Availability

The data and materials generated and/or analysed during the current study are not publicly available, because the participants have not consented for their patient data to be transferred to additional parties.

## Abbreviations

αSMA: α-Smooth muscle actin
COX2: Cyclo-oxygenase 2
HUH: Helsinki University Hospital
ICA: Internal carotid artery
LEC: Lymphatic endothelial cell
LYVE-1: Endothelial hyaluronan receptor 1
MMP-9: Matrix metalloproteinase 9
MPO: Myeloperoxidase
Prox1: Prospero homeobox protein 1
SAA: Serum amyloid A
sIA: Saccular intracranial aneurysm
SMC: Smooth muscle cell
VEGFR-3: Vascular endothelial growth-factor receptor 3
