# Supplementary material for: Lymphatic vessels are present in human saccular intracranial aneurysms
Source: Acta Neuropathol Commun. 2022 Sep 5;10:130. doi: 10.1186/s40478-022-01430-8 (PMC9446758; doi:10.1186/s40478-022-01430-8)
Supplement: Supplementary file 1 — Additional file 1: Table S1. [file 40478_2022_1430_MOESM1_ESM.docx]

**SUPPLEMENTAL TABLE 1.** Primary antibodies used in the immunostainings.

| **Antigen*** | **Function** | **Antibody clone** | **IgG subclass** | **Manufacturer **** | **Concentration (mg/l) or final dilution** |
| --- | --- | --- | --- | --- | --- |
| **LYVE-1** | Cell surface receptor for hyaluronan. Enhances dendritic cell entry into the lymphatic capillaries. | - | Goat polyclonal | R&D Systems | IHC: 2  IF: 4 |
| **Podoplanin** | Cell surface receptor, which binds to C-type lectin-like receptor 2 on platelets. Plays a role in lymphatic separation from the blood vasculature. | D2-40 | Mouse monoclonal IgG1 | Dako | IHC: 0.285  IF: 0.57 |
| **VEGFR-3** | Cell surface receptor for vascular endothelial growth-factor C. | #54703 | Mouse monoclonal IgG1 | R&D Systems | IHC: 10  IF: 10 |
| **Prox1** | Transcription factor, which indicates beginning of the differentiation of lymphatic structures. | - | Goat polyclonal | R&D Systems | IHC: 2 |
| **αSMA** | An actin isoform, which is abundant in vascular smooth muscle cells and contributes to cell-generated mechanic tension. | 1A4 | Mouse monoclonal IgG2 | Sigma-Aldrich | IF: 5 |
| **CD31** | Cell-surface glycoprotein widely used as a marker for endothelial differentiation. Also known as PECAM-1. | JC70A | Mouse monoclonal IgG1 | Dako | IHC: 200 |
| **CD34** | A transmembrane glycoprotein expressed on e.g. endothelial cells and used as a marker for vascular neovessels. | QBEnd/10 | Mouse monoclonal IgG1 | Novocastra | IHC: 12 |
| **Mast cell tryptase** | Protein sectered and stored by mast cells. | AA1 | Mouse monoclonal IgG1 | Dako | IHC: 1:500 |
| **Mast cell chymase** | Protein sectered and stored by mast cells. | CC1 | Mouse monoclonal IgG1 | Serotec | IHC: 1:500 |
| **CD3** | T-cell co-receptor, which is involved in activating cytotoxic T-cells and helper T-cells. | F7.2.38 | Mouse monoclonal IgG1 | Dako | IHC: 1:500 |
| **CD68** | Transmembrane glycoprotein highly expressed by cells in the monocyte lineage. | EBM11 †  KP1 ‡ | Mouse monoclonal IgG1 | Dako †  Thermo Fisher Scientific ‡ | IHC: 2.4  IF: 10 |
| **CD163** | A scavenger receptor for haptoglobin-hemoglobin complexes, which are expressed by monocyte/macrophage-line. | 5C6-FAT | Mouse monoclonal IgG1 | Novus Biologicals | IHC: 20 |
| **Glycophorin A** | Sialoglycoprotein of the human erythrocyte membrane. | JC159 | Mouse monoclonal IgG1 | Novus Biologicals | IHC: 2 |
| **SAA** | A family of apolipoproteins, which are elevated in an acute-phase response (Eklund et al., 2012). | MC1 | Mouse monoclonal IgG1 | Dako | IHC: 1:100 |
| **COX2** | An enzyme responsible for the formation of prostanoids, which are released in high amounts locally at the site of inflammation (Mitchell et al., 1999). | CX-294 | Mouse monoclonal IgG1 | Dako | IHC: 1:100 |
| **MPO** | A lysosomal enzyme expressed mainly in neutrophils and to a lesser degree in monocytes. | A0398 | Rabbit polyclonal | Dako | IHC: 1.8 |
| **MMP-9** | A protein involved in the breakdown of extracellular matrix. | 4H3 | Mouse monoclonal IgG1 | Novus Biologicals | IHC: 0.2 |
| **Apolipoprotein A-I** | A major protein component of the high-density lipoprotein complex, which enables the reverse cholesterol transport. | 1C5 | Mouse monoclonal IgG1 | Monosan | IHC: 0.01 |
| **Oxidized LDL (hydroxynonenal)** | Oxidization of low-density lipoproteins plays a key role in the development of the early atherosclerotic lesions. | HNE | Guineapig polyclonal | (33) | IHC: 1:300 |
| **Adipophilin** | A protein on the surface of intracellular lipid droplets and used as a marker of intracellular lipid accumulation. | AP125 | Mouse monoclonal IgG1 | R&D Systems | IHC: 1:10 |

* LYVE-1 (lymphatic vessel endothelial hyaluronic acid receptor-1), VEGFR-3 (vascular endothelial growth factor

receptor 3), Prox1 (prospero-related homeobox 1), αSMA (alpha-smooth muscle actin); CD (cluster of

differentiation), SAA (serum amyloid A), COX2 (cyclo-oxygenase 2), MPO (myeloperoxidase), MMP-9 (matrix

metalloproteinase-9),

** Dako, Glostrup, Denmark; R&D Systems, Minneapolis, MN, USA; Thermo Fisher Scientific, Carlsbad, CA, USA;

Sigma-Aldrich, St. Louis, MO, USA; Novus Biologicals, Littleton, CO, USA; Serotec, Oxford, UK; Novocastra,

Newcastle upon Tyne, UK; Monosan, Uden, The Netherlands

IHC: immunohistochemical staining

IF: immunofluorescence staining
