# Supplementary figures and images for: Lymphatic vessels are present in human saccular intracranial aneurysms
Source: Acta Neuropathol Commun. 2022 Sep 5;10:130. doi: 10.1186/s40478-022-01430-8 (PMC9446758; doi:10.1186/s40478-022-01430-8)

**SUPPLEMENTAL FIGURE 1**

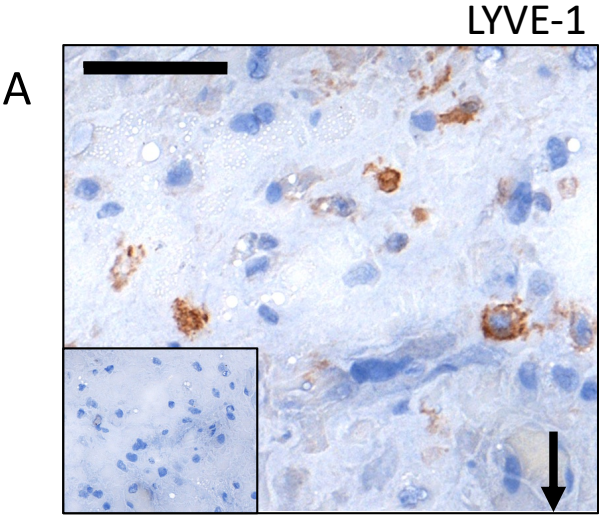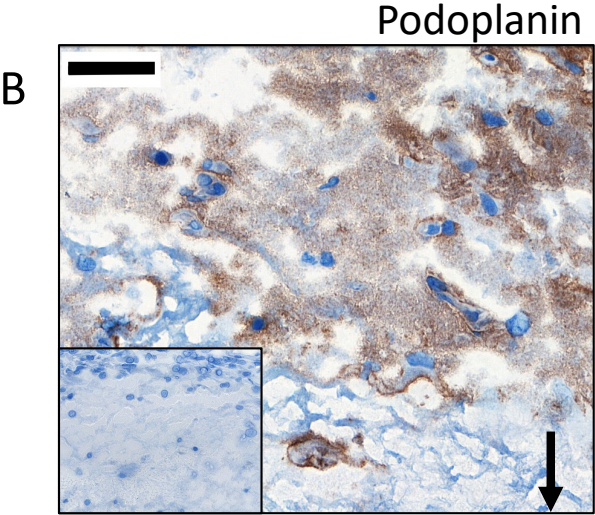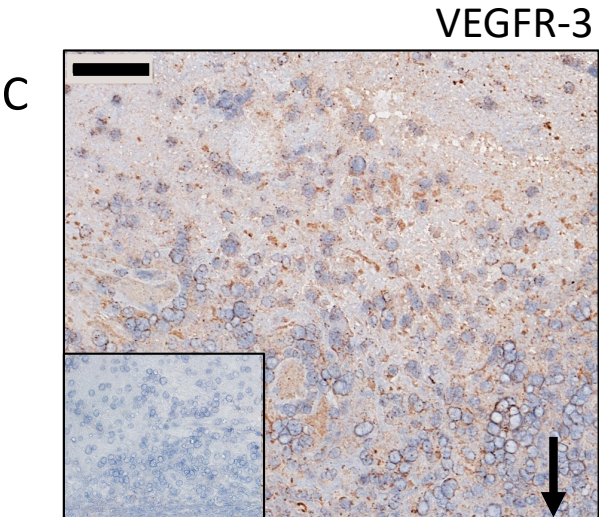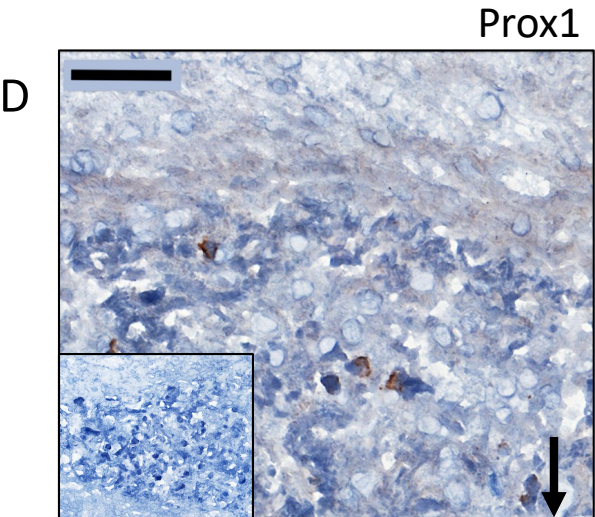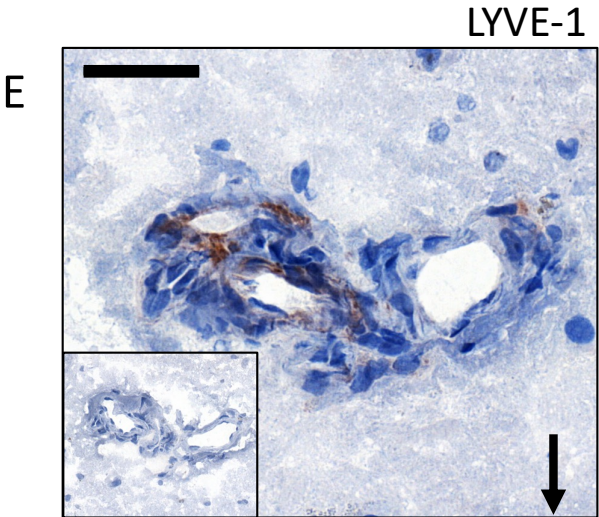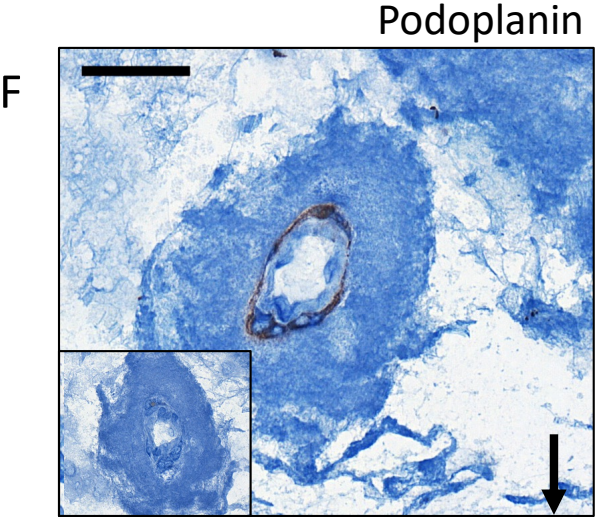

Supplement: Supplementary file 2 — Additional file 2: Fig. S1. Representative images of thrombus in the saccular intracranial aneurysm (sIA) presenting immunohistochemical staining for LYVE-1 (A), podoplanin (B), VEGFR-3 (C), and Prox1 (D). Ring-shaped structures of immunohistochemical staining positive for LYVE-1 (E) and podoplanin (F), i.e., lymphatic vessels, in the sIA thrombus. The negative controls are shown as insets. Black arrows point down towards the lumen. Scale bar: 50 μm. Positive staining is brown. Haematoxylin background staining. [file 40478_2022_1430_MOESM2_ESM.pdf]
